# The effect of parental psychological control on children’s peer interactions in China: the moderating role of teachers’ emotional support

**DOI:** 10.3389/fpsyg.2024.1297621

**Published:** 2024-01-30

**Authors:** Ronghui Chen, Shujuan Li, Siying He, Jin Yan

**Affiliations:** Faculty of Education, Northeast Normal University, Changchun, China

**Keywords:** psychological control, peer interactions, young children, teacher support, China

## Abstract

**Background:**

Peer interactions are critical to young children’s social development, and proximal-system has a direct influence on personal growth. The study aims to analyze the relationship between parental psychological control and young children’s peer interactions, as well as the moderating role of teachers’ emotional support in this association.

**Methods:**

A total of 241 children aged 3–6 years, alongside their parents, and 27 teachers, participated in the study. Teachers reported children’s peer interactions whilst parents reported their psychological control. The level of teachers’ emotional support was co-coded by two researchers.

**Results:**

The results of the study indicated that parental psychological control was significantly and negatively related to young children’s peer interactions; teachers’ emotional support was significantly and positively related to young children’s peer interactions; the cross-level moderating effect validates our hypothesis that teachers’ emotional support has a moderating effect between parental psychological control and young children’s peer interactions, buffering the impact of parental psychological control on young children’s peer interactions.

**Conclusion:**

These findings expand our comprehension of the association between parental psychological control, teachers’ emotional support, and young children’s peer interactions, and provide guidance for integrating the components of the proximal system and devising interventions to establish a home-school harmony environment that fosters children’s social development.

## 1 Introduction

Children begin to establish and maintain social relationships with their peers as well as adults in their environments during early childhood. Peer interactions not only helps children develop their social skills (Ladd, 2005), but also has an important effect on their cognition, emotion and social adaptability (Groh et al., 2014). Research has found that early interactions with peers contribute to the formation of children’s internal schemes of social relations and also provide them with opportunities to directly and indirectly learn how to interact with others, what socially acceptable behaviors are like, and how to appropriately regulate their behaviors to achieve social goals (Beauchamp and Anderson, 2010). Thus, positive peer interactions are generally associated with reduced aggressive behaviors and increased prosocial behaviors, more sociability, communication, and assertiveness (Fantuzzo et al., 2005; Girard et al., 2011; Booren et al., 2012; Acar et al., 2015). Conversely, experiencing peer interactions problems can also reduce children’s cognitive and executive function levels, leading to cognitive impairment and social dysfunction (Zhang et al., 2022), which in turn leads to withdrawal and antisocial behavior in children (Bulotsky-Shearer et al., 2014; Holmes et al., 2016). It can be seen that peer interactions, as an important way to establish social relations in early childhood, has a significant impact on children’s social adaptations and future development.

Peer interactions is a proximal process (Lin et al., 2016), in which significant others, such as parents and teachers, have an important influence on children’s social development (Thompson et al., 2003; Denham et al., 2007). Psychological control is an important dimension to measure the quality of parenting, which refers to parenting behaviors that parents use to intrude and control children by inducing children’s guilt (Schaefer, 1965). Prior studies have found that high levels of parental psychological control not only lead to internalized problems such as anxiety (Pettit et al., 2010; Settipani et al., 2013), depression (Soenens et al., 2005, 2012), and low self-esteem (Bireda and von Krosigk, 2015) in children, but also reduce their self satisfaction (Park and Han, 2020), disrupt their regulatory ability, and trigger aggressive behavior (Koçak et al., 2017), which has a certain negative impact on children’s externalized behavior. Besides parents, teachers’ supportive behavior also has a positive effect on children’s social development. In early childhood, children who are more encouraged by their teachers will have better teacher-student relationships, academic and social development (Silver et al., 2005). Children who experienced higher levels of emotional and classroom quality in kindergarten exhibit better social skills and problematic behaviors in kindergarten and first grade, compared to those who do not experience such high-quality classrooms (Broekhuizen et al., 2016). In addition, the study has points out that teacher support or high quality teacher-student relationships serve as a buffer in the association between negative parenting and child psychological adjustment (Deng et al., 2021).

Previous studies have investigated the associations between teachers, family and children’s social development, separately. However, most works were conducted in the family or school micro-system. There is a lack of research that analyzes the relationships and mechanisms between parental psychological control, young children’s social development, and teachers within the dual system of the family and school. Thus, this study aims to investigate the impact of parental psychological control on young children’s peer interactions, and whether emotional support from teachers moderates this relationship. This will provide a basis for the formation of a home-school synergy that jointly promotes children’s social development.

### 1.1 Parental psychological control and children’s peer interactions

Family is the most central micro-system for children development. Accumulating studies have investigated the link between parenting styles and children’s social development (Bronfenbrenner,1989; Chen et al., 1997; Ren and Pope Edwards, 2015). Psychological control refers to parenting behaviors that intrude upon children’s thoughts and feelings, and has been characterized as used by parents who excessively implement manipulative parenting techniques, such as guilt-induction, shaming, and love withdrawal. As psychological control is thought to inhibit children’s development of a secure sense of self, it would lead to disturbances in psychological functioning (Barber and Harmon, 2002; Soenens and Vansteenkiste, 2010). Studies have found that parental psychological control forces children to comply with their parents’ needs and wishes rather than their own, which interrupts children’s need satisfaction (Deci and Ryan, 2000; Soenens and Vansteenkiste, 2010), undermines their self-control (Nanda et al., 2012). Similarly, some research suggests that parental psychological control can make children overly dependent on others (Aunola et al., 2013). This may have a negative impact on children’s self-schema (Mcleod et al., 2007) and psychological autonomy (Oudekerk et al., 2015). As a result, the child may become overly compliant in their peer interactions and may even become a victim of bullying (Chen et al., 2021). Research has shown that parental psychological control predicts a negative impact on children’s friendship quality, resulting in increased levels of loneliness (Soenens et al., 2008). Typically, parents with higher levels of psychological control tend to be less sensitive and responsive to children’s needs, result in insecure attachments with children, deprive children of a ‘safe base’ for exploring new tasks and developing new relationships, and consider themselves untrustworthy and unsupportive (Cook et al., 2012). Furthermore, children who experience psychological control from their parents may feel insecure in their negative attachment relationships and may resort to using the same methods to control peers (Michiels et al., 2008).

Although the concept and research of psychological control originated in Western countries, Chinese parents generally exhibit higher levels of psychological control compared to their Western counterparts (Shek, 2007; Scharf and Goldner, 2018). This can be attributed to the influence of Confucianism, which emphasizes hierarchical and harmonious relationships and has had a significant impact on Chinese culture. In line with this philosophy, parents hold a position of authority (Hoang and Kirby, 2020), and children are expected to show absolute obedience toward their parents as a sign of respect. Additionally, when parents intervene excessively in their children’s lives, it is often perceived as an expression of love. To maintain hierarchy and harmony, Chinese parenting styles tend to include psychological control as a characteristic feature. As parental psychological control is prevalent in China, there is a growing interest in investigating it in Chinese culture. Several studies have shown that parental psychological control is positively associated with children’s internalizing and externalizing behavioral issues in Chinese culture (Wang et al., 2007; Li et al., 2018). Children who have parents with psychological control tend to report higher levels of depression (Bullock et al., 2018), loneliness (Li et al., 2019), and social anxiety symptoms (Luebbe et al., 2018). Additionally, a positive association has been found between parental psychological control and aggression (Chen et al., 2020) as well as antisocial behaviors (Sun et al., 2017). Some studies also revealed that when a mother has a high degree of psychological control, her children are at an increased risk of developing poor peer relationships. When the level of psychological control exercised by the mother is high, social avoidance significantly and positively predicts children’s antisocial behaviour and peer rejection (Zhao and Wang, 2010; Zhu et al., 2020).

Early childhood experiences can significantly affect future development. A review of the existing literature shows that studies have primarily focused on the effects of parental psychological control on children’s social development during mid-childhood or adolescence. It remains uncertain the relationship between parental psychological control and young children’s peer interactions. Clarifying this association in preschool years can provide vital information for theoretical and cross-cultural studies on children’s early social development and family education.

### 1.2 Teachers’ emotional support and children’s peer interactions

School is another important micro-system factor for child development, and teachers are the primary adult figures within this micro-system. Teachers’ emotional supportive behaviors, including showing concern, respect, understanding, encouragement, and trust, can positively impact children’s autonomy, efficacy, and social development (Fredricks et al., 2004; Skinner et al., 2008). The study showed that the greater teacher support children felt, the higher the gain in positive functioning (grades, social initiative with teachers and peers), and the lower the increase in negative functioning (deviant peer association, parent–child conflict, depression), This was also associated with stronger interpersonal skills (Barber and Olsen, 2004). In the preschool years, emotional interactions between teachers and children are key to the social development of young children. Positive attention, encouragement, and praise from teachers during interactions with children enhance the confidence of left-behind children in interpersonal communication (Lei and Li, 2020). Teachers’ warm and sensitive behaviour can enhance the self-confidence and efficacy of young children in interacting with others. Conversely, teachers’ insensitive and unresponsive behaviour may lead to maladaptation in both self and interpersonal relationships (Verschueren and Koomen, 2012). When teachers create a warm and caring atmosphere in the classroom, children develop a sense of security and trust, which makes children willing to participate in group activities and actively interact with their peers (Downer et al., 2010; Ruzek et al., 2016). Moreover, children’s perception by their peers can be influenced by teacher’s expectations and approval.

Research has confirmed the beneficial effects that teacher support and and emotional interactions on children’s social interaction skills. However, there is a relative scarcity of studies investigating the correlation between teachers’ emotional support and young children’s peer interactions. In sum, teachers who offer emotional support to their students can strengthen their bond, and a positive teacher-student relationship may help mitigate social adjustment difficulties in children. As a consequence, it is necessary to explore the correlation between teachers’ emotional support and young children’s peer interactions. This can help establish cordial teacher-child relationships and foster the social development of children.

### 1.3 The moderating effect of teachers’ emotional support

According to the risk and protective factor framework, child development is the dynamic interplay between risk and protective factors (Deng et al., 2021). Whereby risk factors predispose children to negative developmental outcomes, and protective factors increase resilience and decrease the likelihood of negative outcomes (Masten, 2001; Wang et al., 2013). As an essential part of the proximal process, the teacher not only plays a direct role in the child’s development, but also acts as a protective shield against family risks that may have a negative impact on children. Some research has found that positive relationship with teachers compensated for unsafe relationships with parents. The warmth and trust provided by teachers eased conflicts between parents (Lynch and Cicchetti, 1997), buffered children with negative family experiences from misbehavior (Wang et al., 2013), and reduced the anxiety caused by mothers’ psychological control (Deng et al., 2021).

To date, the majority of current research on parental psychological control and children’s peer interactions have focused on the negative effects of family micro-systems, but there is a dearth of research on protective factors that mitigate these harmful effects. Considering the positive impact of teachers’ emotional support on children’s peer interactions and the protective role of positive teacher-child relationships between children and family risks, this study aims to examine whether teachers’ emotional support can moderate the relationship between parental psychological control and children’s peer interactions.

### 1.4 The present study

Warm and supportive positive parenting can strongly guarantee children’s social development, whereas negative parenting practices, such as abnormal parent–child interactions and negative parental control, are the primary risk factors for the psychological and social issues of children. In China, at the adolescent stage, high levels of psychological control by parents have become increasingly prevalent due to the influence of hierarchical authority ideology and social competition pressure. With growing awareness of the significance of early childhood development and increasing parental investment in early education, it is crucial to examine the psychological control of parents with young children. While previous studies has highlighted the negative effects of parental psychological control on young children’s social adaptation and the positive effects of teachers’ emotions on their social development, the relationship between these factors remains largely unexplored, particularly with regard to their interaction within proximal processes. Based on relevant theories and literature evidence, we formulated a hypothetical relationship model. The research hypotheses were as follows:

*H1*: Parental psychological control is negatively correlated with young children’s peer interactions.

*H2*: Teachers’ emotional support is positively with young children’s peer interactions.

*H3*: Teachers’ emotional support plays a moderating role between parental psychological control and young children’s peer interactions.

## 2 Methods

### 2.1 Participants

To identify participants, the researcher communicated with kindergarten administrators, announced the research, and solicited volunteers. In China, early childhood teachers are almost exclusively female; therefore, the classroom teachers who volunteered to participate in this research was all female. Subsequently, the consent letters was sent the parents by the classroom teachers. The participants included 241 young children (aged 3–6) from 5 preschools in China, along with their pater or mater and 27 classroom teachers. Among the sample of children, there were 128 boys (53.10%) and 113 girls (46.90%) and 19.90% were aged 3–4, while 33.60% were aged 4–5 and the remaining 46.50% were aged 5–6. Additionally, the survey included 170 mothers (70.50%) and 71 fathers (29.50%), who were actively involved in childcare and had frequent interactions with their children. Among the surveyed parents, 48.10% had a high school education or below, 32.80% had completed junior college, and 19.10% held a bachelor’s degree or higher. Furthermore, the study involved 27 female teachers, of whom 8 (29.63%) had less than 3 years of experience, 13 (48.15%) had worked for three to 5 years, and 6 (22.22%) possessed more than 5 years of experience.

### 2.2 Procedure

We conduct data collection based on the following processes and criteria. Step 1: The measurement of children social competence were implemented one by one by the classroom teachers. The whole evaluation process complete in 3 months. Step 2: The teachers and the researchers jointly distributed the questionnaires of the psychological control assessment to the parents. It took 1 month to collect all the questionnaires. Step 3: The teachers’ emotional support was recorded and evaluated by two coding team members (both with systematic, targeted training). In the specific operation process, the interactive scenes between teachers and children were recorded and selected on video by means of collective teaching activities, area activities, life activities and outdoor activities in the daily life of the kindergarten. During the coding process, two coding team members watched the whole contextual video carefully together and discussed it. The edited recording units were all about 30 min, and at least 6 observation units were collected for each teacher. The entire recording, excerpt and coding process took about 6 months. The Kendall concordance coefficient encoded by the two researchers was 0.97.

### 2.3 Measurement

#### 2.3.1 Children’s peer interactions measurement

Children’s peer interactions were measured with *Children*’*s Social Competence with Peers Scale for 3–6 year old children* (CSCPS) (Li, 2008). The scale was designed and developed by a Chinese scholar in 2008 according to the *peer interactions scale* (PIS) (Williams et al., 2007) and *Teacher-rated Peer Competence Scale (TPCS)* (Howes et al., 1988). The scale consists 20 items covered by four dimensions: social initiative, verbal and non-verbal ability, prosocial behavior and social disorder. The participants rated their responses on a 5- point Likert scale (form 1 = Never to 5 = Always). Confirmatory factor analysis (CFA), CFI = 0.968, TLI = 0.957 were all greater than 0.90; *χ*^2^/df = 1.860 < 3, RMSEA = 0.060 < 0.08, SRMR = 0.038 < 0.05. Therefore, the scale was suitable for use in the current research. Cronbach’s alpha was 0.855, 0.781, 0.765, and 0.882, respectively, for each dimension, the total internal consistency coefficient of the scale was 0.898.

#### 2.3.2 Parental psychological control measurement

Parental psychological control was measured with the Chinese version of the Parental Control Scale for 3–5 year old children (Chen, 2007). The scale consists 34 items covered by 13 dimensions: Love withdrawal, Control, Control by guilt induction, Suppression of aggression, Control by anxiety induction, Negative affect, Protectiveness, Supervision, Emotional support, Rational guidance, Parental independence, Expression of affect, encouragement of independence. The participants rated their responses on a 5-point Likert scale (form 1 = Strongly disagree to 5 = Strongly agree). Confirmatory factor analysis (CFA), CFI = 0.993, TLI = 0.991 were all greater than 0.90; χ^2^ / df = 1.0539 < 3, RMSEA = 0.015 < 0.08, SRMR = 0.035 < 0.05. As a result, the scale was suitable for use in the current research. Cronbach’s alpha was 0.699–0.898, respectively, for each dimension, the total internal consistency coefficient of the scale was 0.908.

#### 2.3.3 Teachers’ emotional support measurement

Classroom Assessment Scoring System (CLASS) was a common tool for evaluating the quality of classroom in kindergarten recently (Pianta, 2012; Ishimine and Tayler, 2014). Emotional Support which is a part of CLASS was adopted for evaluating teacher in the study. The scale consists 16 items covered by four dimensions: Positive climate, Negative climate, Teacher sensitivity and Regard for student perspectives. The observers assessed on a 7-point Likert scale, scores of 1–2 indicate low on this dimension, 3–5 indicate mid-range, and 6–7 indicate high. Cronbach’s alpha encoded by the two researchers was 0.913, 0.807, 0.845, and 0.705 respectively, for four dimensions. The total internal consistency coefficient of the scale was 0.907.

### 2.4 Data analysis

Descriptive statistical analysis and Pearson’s correlation analysis were used to determine the performance level of each variable on different demographic factors, as well as correlations among variables, using the SPSS 26.0. Furthermore, parents’ psychological control and teachers’ emotional support as the relevant variable of peer social competence, which can be described as a nested relationship because they were influencing factors from different levels. Therefore, this study retained these predictors at the appropriate level of analysis using the Mplus 8.3. The effects of predictors at different levels on the outcome variables were analyzed using Multilevel Structural Equation Modeling to test for moderating effects.

## 3 Results

### 3.1 Common method deviation analysis

Since the parental control data were derived from self-reports, there may be common methodological bias and reduce the validity of the study. Harman single factor method was used to test the common method bias before data analysis. The results showed that all 13 variables had a value >1. The variation explained by the first unrotated factor was 26.32%, which is <40 percent, indicating that there is no substantial common method bias in this study.

### 3.2 Preliminary analyses

Descriptive statistics and correlations for all observed variables are shown in Table 1.

**Table 1:** Descriptive statistics and correlations of observed variables.

Variables	1	2	3	4	5	6	*M (SD)*
1.Child gender							0.469 (0.500)
2.Child age	−0.011						2.266 (0.772)
3.Parents gender	0.042	−0.108					0.710 (0.457)
4.Parents education	−0.024	0.018	−0.055				1.709 (0.768)
5.Paternal psychological control	−0.202**	0.302**	−0.186**	−0.133*			2.480 (0.574)
6.Teachers’ emotional support	0.002	0.208**	−0.129*	0.043	0.076		5.043 (0.162)
7.Children peer interactions	0.167**	0.312**	−0.05	0.244**	−0.339**	0.176**	2.873 (0.693)

*p < 0.05; **p < 0.01; ***p < 0.001.

Firstly, children exhibit a medium level of peer interactions (*M* = 2.873), and there exist discernible variations between gender and age. Girls demonstrate a higher aptitude for peer interactions (*M* = 2.996, SD = 0.663) than boys (*M* = 2.765, SD = 0.703), and as children age, their proficiency in this area develops accordingly. Therefore, gender and age were considered for subsequent analysis. Secondly, the degree of psychological control exerted by parents is lower than medium (*M* = 2.480). Overall, fathers display a higher level of psychological control than mothers. Additionally, there were significant differences in the degree of psychological control concerning the gender and age of children and the educational level of the parents. Specifically, boys experienced greater psychological control from their parents than girls (*t* = 3.196, *p* < 0.01), and parents with lower educational levels exerted higher levels of psychological control. Consequently, we also controlled for effects of parents’ gender and educational levels. Thirdly, teachers’ emotional support is at the middle and upper level (*M* = 5.043), and there is no substantial variation in the emotional support given by teachers in different types of activities.

Correlation analysis of the three variables (as displayed in Table 1) demonstrated a significant negative correlation between PPC and CPI (*r* = −0.339, *p* < 0.01), and a positive correlation between TES and CPI (*r* = 0.176, *p* < 0.01). Typically, the strength of the linear association between two variables is initially evaluated by the correlation coefficient. If the correlation reaches a statistically significant level, the linear relationship has significance and further regression analysis can be carried out for prediction and interpretation.

### 3.3 Moderating role of TES in PPC and CPI

The Multilevel Structural Equation Modeling (MSEM) is used to evaluate measurement and structural models across multiple levels of the analysis in situations where nesting is present. In this study, we utilized MSEM to evaluate children’s peer interactions at both the individual-within and group-between levels.

First, a null model analysis was performed to examine the intraclass correlation coefficients (ICC), in order to determine the presence of between-group variance of model variables and to check the significance of between-level residual variance. The results of the null model analysis showed that the between-group variance t_00_ was 0.058, the within-group variance σ^2^ was 0.042 and the ICC was 0.122, surpassing the threshold of 0.06. Therefore, inter-group variation must not be disregarded and a multilayer analysis becomes imperative.

Secondly, we investigated the extent to which parental psychological control and were associated with children’s peer interactions. We used it as a predictor variable in Model 1 and controlled other main effects terms (e.g., gender, age). The results showed that the effect of parental psychological control was significant and negative (r = −0.456, *p* < 0.05), indicating that parental psychological control had a negative predictor of children’s peer interactions.

Next, to ascertain whether the relationships between the key study variable teachers’ emotional support and children’s peer interactions, the group mean of parental psychological control in Model 2 was placed into Model 3 as the predictor variable. Due to the analysis of cross-level data, Random Coefficient Prediction (RCP) was considered to make it more appropriate for multilevel analysis to test the hypotheses (Preacher et al., 2016). The results of the multilayer regulation effect test showed that the cross-layer regulation effect was significant (*r* = 1.847, SE = 0.462, *p* < 0.001). Results for the multilevel models were depicted in Table 2.

**Table 2:** Predictors of peer interaction with multilevel weights.

Predictors	Model 1		Model 2		Model 3	
	***β***	*SE*	***β***	*SE*	***β***	*SE*
Child gender	0.099	0.101	0.235	0.128	0.081	0.104
Child age	0.271*	0.118	0.242***	0.035	0.189**	0.064
Parents gender	−0.114	0.064	−0.015	0.120	−0.114	0.061
Parents education	0.132***	0.039	0.203***	0.051	0.107*	0.053
Children peer interactions	2.118^***^	0.273	1.879***	0.165	2.362***	0.073
Paternal psychological control	−0.456*	0.189			−0.436**	0.148
Teachers’ emotional support			0.462***	0.143	0.601***	0.115
Paternal psychological control×teachers’ emotional support					1.847***	0.462

*p < 0.05; **p < 0.01; ***p < 0.001.

**Figure 1:**
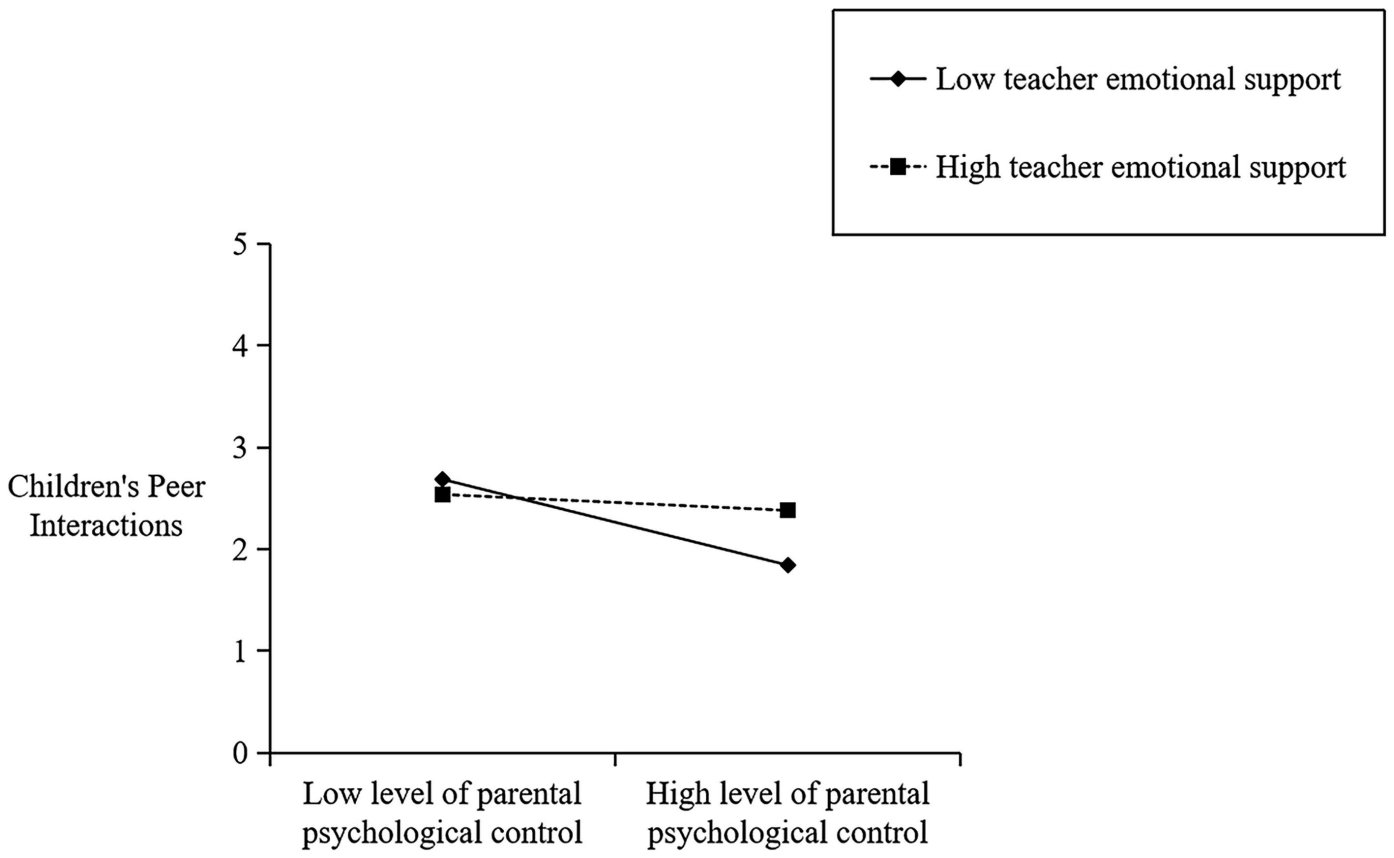
Plot of cross-level interaction: teacher emotional support as a moderator of the effect of parental psychological control regressed on children’s peer interactions.

Table 2 also presents the cross-level moderate effect of teachers’ emotional support. By adding teachers’ emotional support variable, the relationship between parental psychological control and children’s peer interactions can be significantly positively moderated (*β* = 1.847, *p* < 0.001). This means that teachers’ emotional support attenuated the negative impact of parental psychological control on children’s peer interactions. Following Aiken and West’s (1991) procedure, the moderation effect was plotted by computing slopes one standard deviation above and below the mean of the moderator. The cross-level moderation effect is presented in Figure 1. The results show that parental psychological control has a significant negative effect on children’s peer interactions (*β* = −0.596, *p* = 0.000) when teachers’ emotional support is low (-1SD), while when teachers’ emotional support is high (+1SD), parental psychological control has no significant effect on children’s peer interactions (*β* = −0.102, *p* = 0.586). It can be seen that teachers’ emotional support plays a certain moderating role between parents’ psychological control and children’s peer interactions.

**Figure 2:**
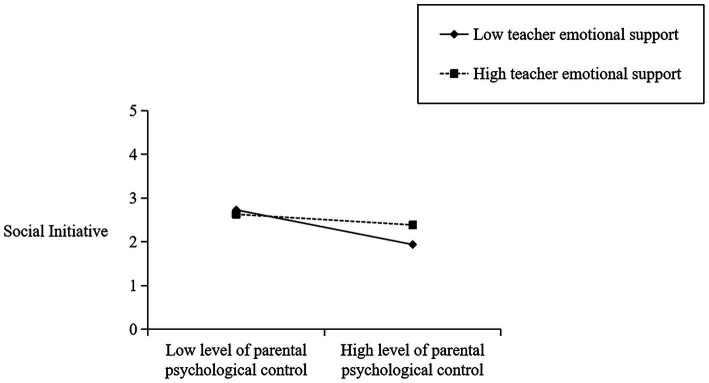
Plot of cross-level interaction: teacher emotional support as a moderator of the effect of parental psychological control regressed on children’s social initiative.

**Figure 3:**
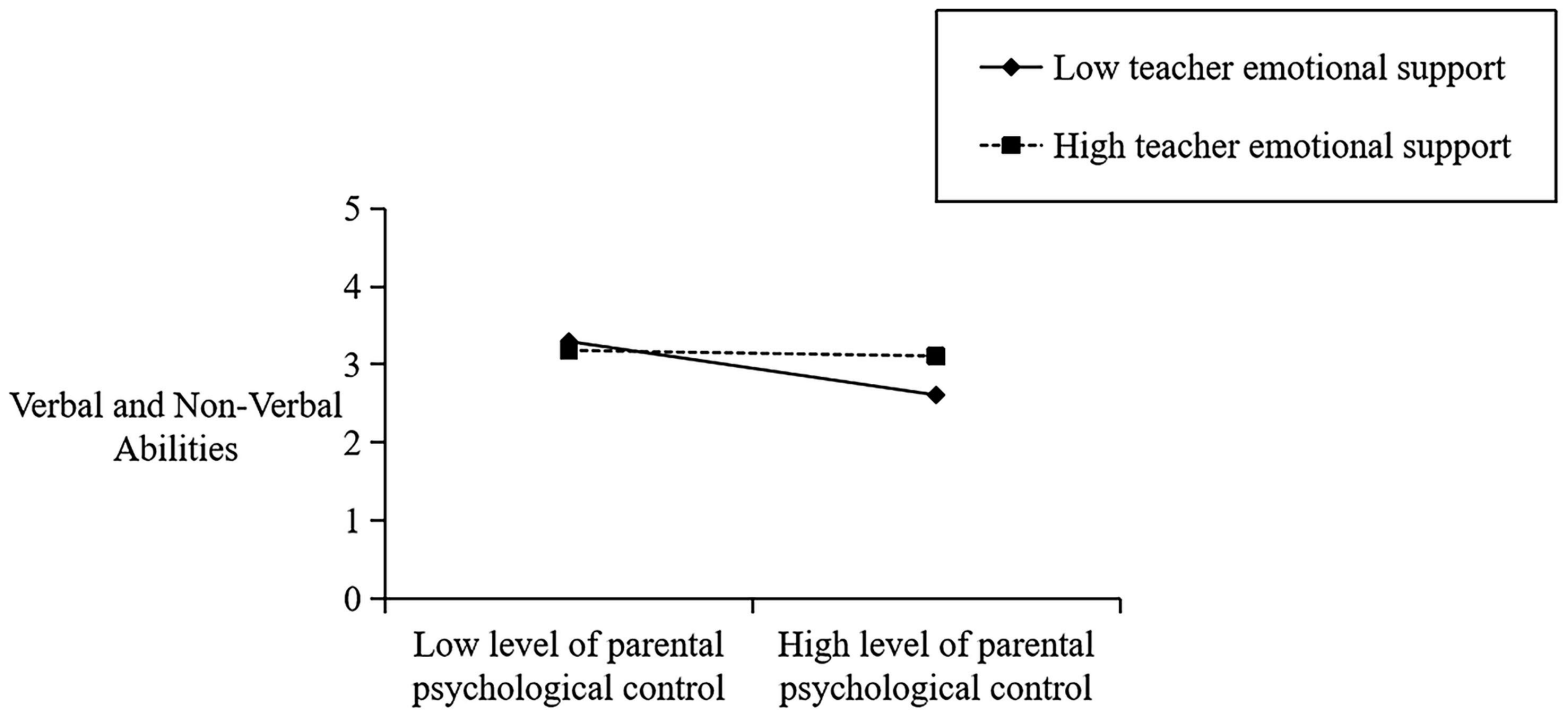
Plot of cross-level interaction: teacher emotional support as a moderator of the effect of parental psychological control regressed on children’s verbal and non-verbal abilities.

**Figure 4:**
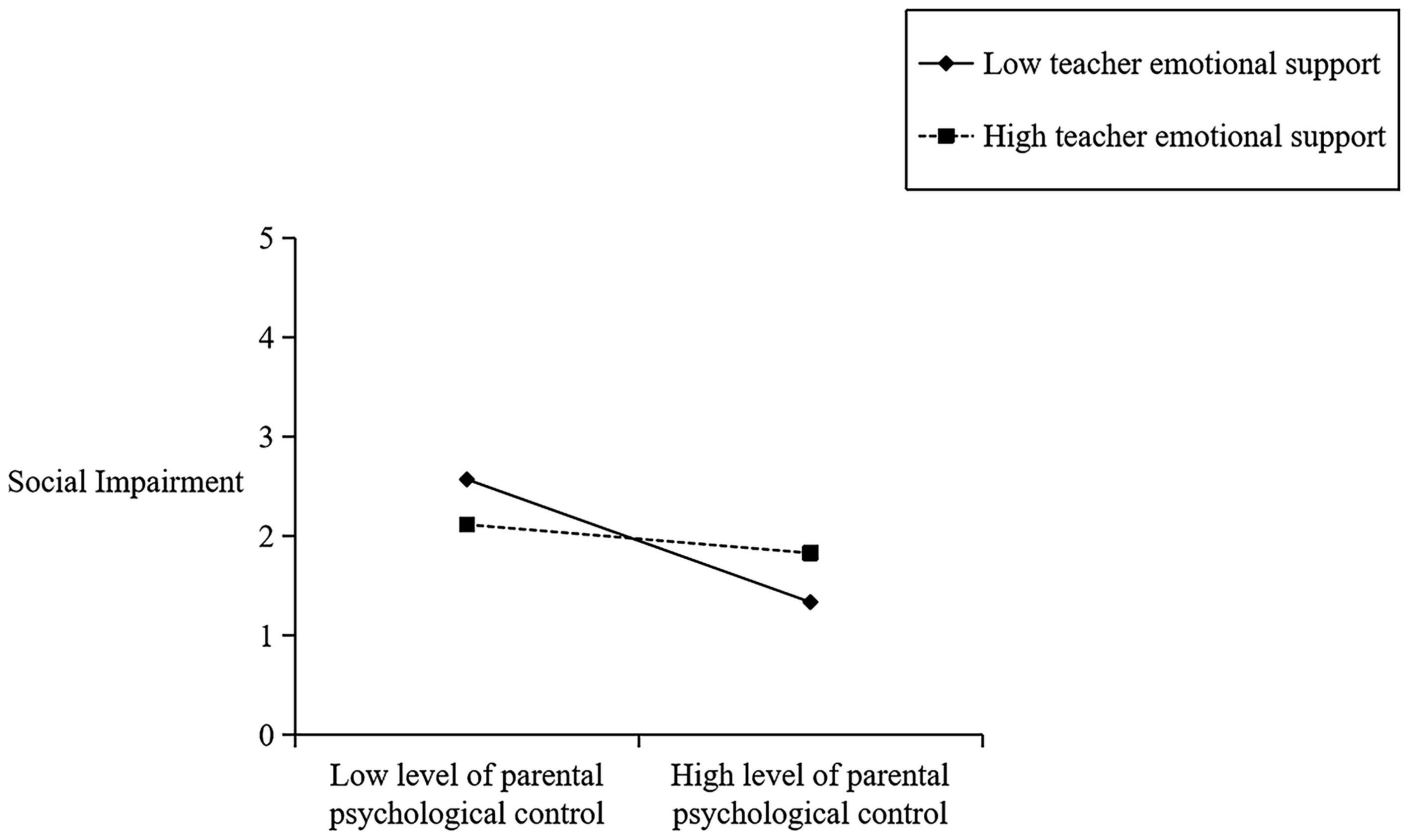
Plot of cross-level interaction: teacher emotional support as a moderator of the effect of parental psychological control regressed on children’s social impairment.

To delve deeper into the regulatory function of teachers’ emotional support in the facets of parents’ psychological control and children’s peer interactions, all variables except gender were standardized. Table 3 displays the cross-level interaction effect of teachers’ emotional support on the specific dimensions of children’s peer interactions. As shown in Table 3, teachers’ emotional support is a crucial predictor of social initiative (*β* = 1.475, *p* < 0.001), revealing that it fosters a positive association between parental psychological control and children’s peer interactions. As depicted in Figure 2, when there is a low level of emotional support from the teacher (-1SD), parental psychological control has a negative impact (*β* = −0.689, *p* = 0.000) on their children’s peer interactions. Conversely, when the level of emotional support from teachers is high (+1SD), parental psychological control does not have a significant effect on children’s peer interactions (*β* = −0.211, *p* > 0.05). In addition, Table 3 also reveals that teachers’ emotional support has a moderating effect on the relationship between parental psychological control and verbal and non-verbal abilities (*β* = 1.650, *p* < 0.001) and social impairment (*β* = 2.548, *p* < 0.001) (see Figures 3,4), but not between parental psychological control and prosocial behaviors (*p* > 0.05).

**Table 3:** Multilevel model (random slope model) of teachers’ emotional support’s moderating role in relations between parental psychological control and children’s peer interactions.

Variables	Social initiative		Prosocial behavior		Verbal and non-verbal abilities		Social impairment	
	***β***	*SE*	***β***	*SE*	***β***	*SE*	***β***	*SE*
Constant	2.510***	0.366	2.121***	0.640	3.050***	0.089	1.965***	0.057
Child gender	0.017	0.112	0.232	0.105	0.025	0.085	0.039	0.211
Child age	0.154***	0.036	0.199	0.268	0.126***	0.023	0.282***	0.043
Parents gender	−0.114	0.086	−0.052	0.089	−0.084	0.070	−0.296	0.181
Parents’ education	0.105	0.056	0.158**	0.050	0.009	0.038	0.101	0.072
Paternal psychological control	−0.450***	0.155	−0.397	0.210	−0.330**	0.117	−0.662***	0.208
Teachers’ emotional support	0.536***	0.183	1.071	0.708	0.594***	0.102	0.059	0.314
Cross-level interaction	1.475***	0.395	2.078	1.255	1.650***	0.393	2.548***	0.548

*p < 0.05; **p < 0.01; ***p < 0.001.

## 4 Discussion

The objective of this study was to examine the association between psychological control exerted by parents and the peer interactions of preschool children in China. Additionally, the study sought to establish whether emotional support provided by teachers could moderate this relationship. According to our findings, children’s peer interactions were linked to both parental psychological control and teachers’ emotional support. Furthermore, our research revealed that teachers’ emotional support can serve as a protective factor against the negative influence of parental psychological control on children’s peer interactions. To our knowledge, this study is the first to reveal the moderating role of teachers’ emotional support in the connection between parental psychological control and children’s peer interactions. This constitutes a valuable addition to our comprehension of the correlation between parental psychological control and peer interactions. Moreover, the findings of our study may be utilized to recognize efficient interventions in order to enhance the relations between home and school, and to augment the level of peer interaction among children.

### 4.1 Relationship between parental psychological control and children’s peer interactions

Our results confirm the existence of parental psychological control in preschoolers and indicate its problematic impact on young children’s peer interactions. These findings corroborate previous research by Chinese scholars showing that negative parental psychological control predicts social withdrawal in children (Cao, 2011) and that maternal psychological control exacerbates negative adjustment in socially avoidant children (Zhu et al., 2020).

The results of several cross-cultural studies can explain these phenomenons. According to the research, there is a prevalent educational model in China that emphasizes parental authority and children’s obedience (Lai et al., 2014), which is influenced by traditional cultural concepts. In this model, Chinese parents tend to exert a high level of psychological control over their children (Wang et al., 2007) and utilize love withdrawal (Wu et al., 2002) and trigger children’s feelings of guilt as a means of moral socialization (Fung and Chen, 2001). It is evident that Chinese traditional family culture and educational ideologies could be a possible factor that motivates the parent–child relationship and psychological control by parents.

Some studies have analyzed the type of parental psychological control, Soenens et al. (2010) proposed and tested the idea that the validity of a distinction between two domain-specific expressions of psychological control, that is, dependency-oriented and achievement-oriented psychological control. Dependency-oriented parental mental control is often manifested by manipulating the attachment bond with children and use their love and care to control their children. Such parents, particularly mothers, may struggle with the separation resulting from their children’s ongoing growth and view the advancement of their children’s autonomy as a potential danger to their parent–child relationship. To prevent their children’s emotional and psychological independence, these parents employ psychological control tactics, such as love withdrawal, which restrict their children’s interactions outside of the family unit. As a result, these children become dependent on their parents for emotional and psychological support (Barber and Harmon, 2002). Parents who are achievement-oriented and press themselves perform highly may behave in a controlling manner toward their children. They demand perfection and high levels of achievement from their children, and may use psychological control to achieve their expectations. Research has suggested that parental environments with a high emphasis on ego-oriented perspectives of achievement may hinder the children’s optimal development and motivation for achievement (McArdle and Duda, 2004).

It has been found that children aged three to 6 years old are at a crucial stage of social development and the establishment of an independent personality. Parents are more likely to exert dependency-oriented psychological control and display overprotective and possessive behaviour toward their children at this stage. When parents interfere excessively in their children’s personal lives without regard for their autonomy, it may lead to tension, anxiety, and over-dependence, and finally, social withdrawal. Grolnick et al. (2007) presented parents who value their children’s performance may attempt to manage their thoughts, feelings, and behaviors to reduce their likelihood of failure. This could occur even if their children are currently performing well, since the risk of future failure is perceived as a threat. Fundamentally, parents may perceive it necessary to enforce instructions on their children, irrespective of the impact on their children’s independence and sense of importance or capability, still resorting to psychological manipulation. Especially in China, where filial piety is highly valued, parents have significant expectations for their children to achieve elite status in order to fulfill their duty of support. However, due to the uneven distribution of social resources, only a select few are able to achieve this. Consequently, social pressure and competition are on the rise, and parental control over children is becoming more intense (Fong, 2004).

In summary, in China, due to competitive social reality and high parental expectations for preschool children development, some parents may underestimate their children’s abilities. They have the tendency to dominate their children in an autocratic way and force them to achieve perfection in their performance to satisfy specific psychological expectations. These parents force their children to do as they say, depriving them of opportunities for independent thinking and exploration, which undermines their self-efficacy and hinders their autonomous psychosocial functions.

According to the principles of attachment and self-determination theories, negative parenting could impair children’s capacities to form the correct expectations of healthy relationships, both at the home and with peers. In this context, parental psychological control can evoke feelings of insecurity in the parent–child relationship, and the resulting relational insecurity can undermine children’s self-assurance in peer interactions, which in turn, may lead to elevated aggression levels. In China, researchers found that high level of psychological control may dent children’s self-worth (Gao et al., 2018), self-reliance and self-esteem, and even harm children’s socioemotional development (Xing et al., 2017). Consistent with previous research in the United States and China, parental psychological control is associated with childhood aggression in Russia (Nelson et al., 2013). In conclusion, both in China and other countries, parental psychological control is a negative parenting style with adverse effects, which needs to take effective intervention measures to reduce its potential risk.

### 4.2 Effect between teachers’ emotional support and children’s peer interactions

This study further found that teachers’ emotional support was significantly and positively associated with children’s peer interactions. It is similar to the findings of Pianta (2012), the high level of teachers’ emotional support in kindergarten classes has a beneficial relationship with children’s peer interactions. Moreover, according to the correlation analysis of each dimension of teachers’ emotional support and children’s peer interactions, it is evident that there exists a significant and positive correlation, particularly pronounced with regards to children’s prosocial behaviors. Mashburn et al. (2008) noted that high-quality emotional support can promote not only language and academic learning but also social and emotional development. The results of Pakarinen et al. (2020) also showed that teachers’ emotional support can enhance children’s social expectations in the classroom, thereby impacting their subsequent prosocial behaviors.

The multiple motivation theory suggests that emotional support from teachers can boost students’ motivation, while students’ stronger beliefs in their abilities can encourage them to form positive relationships with their peers (Skinner et al., 2008). Typically, the beliefs and behaviors of teachers in their classrooms and their care and support for pupils as a whole shape the specific classroom culture by influencing the children’s classroom environment, which in turn influences their peer interactions (Chang, 2003). Emotionally supportive interactions in the classroom provide young children with ample opportunities to practice skills related to peer relationships (Hamre et al., 2013) and create a specific climate that is conducive to the development of children’s social competence and behaviour (Xing et al., 2017). This is because emotionally supportive interactions can serve as a safe base to help children take social risks, such as the risk of rejection in peer interactions (Verschueren and Koomen, 2012). Thus, to introduce the role of teachers in children’s peer interactions, the concept of “invisible hand”is presented as a metaphor to describe the potentially influential on children’s peer relationships and their broader interpersonal growth (Farmer et al., 2011), illustrating emotionally and behaviorally supportive teacher-child interactions is positively associated with children’s social and emotional skills (Broekhuizen et al., 2017). Several longitudinal studies have demonstrated that conflicts between teachers and children during the early stages of preschool and kindergarten correlate with greater internalization difficulties which may arise later in the academic year (Roorda et al., 2014). First-year pupils demonstrated reduced instances of negative and disruptive conduct in classrooms that provided increased high levels of emotional support and evaluation feedback (Wilson et al., 2007), thereby affirming the significance of providing emotional support in the preschool stage.

Our study also found that teachers scored the highest on emotional support in outdoor games, which was consistent with some of the previous research results (Broekhuizen et al., 2017). The reason is during the naturalistic play situation, which is full of interaction and cooperation, teachers have more opportunities to provide support for children to enhance their social skills.

### 4.3 Teachers’ emotional support moderates parental psychological control and children’s peer interactions

The result of the multilayer moderating effect in this study show that teachers’ emotional support has a significant moderated effect between parental psychological control and children’s peer interactions. This finding was consistent with previous research findings. Spjeldnes et al. (2010) investigated the buffering effect of teacher support on the association between interparental conflict about child-rearing issues and preschool children social skills. It was discovered that when children perceive their parents’ high level of psychological control, it is easy to form a low-security parent–child relationship. However, teachers’ emotional support in the classroom environment promotes the development of teacher-child relationship, which compensates for children to some extent to alleviate the negative effects of family adverse factors in children’s social development (Liu et al., 2023).

According to the multiple attachment theory, children can receive varying emotional support and connection from teachers, viewing them as an additional attachment object. This relationship can be seen as an extension of the parent–child attachment relationship, and it also contributes to safeguarding and promoting the social development of children. As a protective factor, emotional support from teachers imparts a positive influence in enhancing children’s social development and proficiency in associating with peers. Additionally, it helps mitigate the negative effects of family risk factors, narrowing the developmental gap among children.

This study further reveals that parental psychological control has a negative effect on children’s peer interactions, and this effect is significant in the low teachers’ emotional support condition. While, in the high teachers’ emotional support condition, this negative effect of parents’ psychological control on children’s peer interactions is not significant. This indicates that high-level teachers’ emotional support can mitigate the negative impact of parents’ psychological control on children’s peer interactions. This result is similar to previous studies on the relationship between children and the psychological development of young children. Li et al. (2014) suggested that teacher-child relationship showed moderating role in the associations between mother–child relationship and social adaptive behaviors especially for migrant children. Lower teacher-child conflict buffered the negative effects of mother–child conflict on migrant children’s peer interactions; higher teacher-child closeness attenuated the negative effects of mother–child conflict on migrant children’s internalizing problem behaviors. It’s noteworthy that in another study, although teacher-child relationship can mitigate the negative effect of family cumulative risk on children’s resilience, it still plays a rather limited function like a drop in the ocean (Liu et al., 2023). Therefore, the extent to which teachers’ emotional support moderates its effects may also be limited by the extent of family cumulative risk. Additionally, this study explored the relationship between teachers’ emotional support, parental psychological control, and children’s peer interactions. The findings suggest that when parents exhibit high levels of psychological control, teachers’ emotional support for moderating children’s prosocial behavior may not have a significant impact. This highlights the direct and profound influence of family factors on children’s social development, particularly the need to address parents’ psychological control issues.

In conclusion, our study provides evidence that high levels of teachers’ emotional support can moderate the impact of high parental psychological control on children’s peer interactions to some extent. However, as parental psychological control strengthens, the extent to which teachers’ emotional support can moderate it requires further confirmation.

## 5 Conclusion

Based on the ecosystem theory and the risk-protective factor framework, this study identifies the factors that impact young children’s peer relationships. The study developed a moderating model to explore the influence of parental psychological control and teachers’ emotional support on their peer interactions. The findings indicate that excessive parental psychological control poses a risk to young children’s peer interactions. Moreover teachers are integral to the development of young children, their positive emotional support can offset the negative impacts of parental psychological control and bolster healthy peer interactions.

From this point of view, the results of this paper may have important practical significance. First, parents and educators must recognize that home and school are the primary environments where children develop, and that the actions and words of influential individuals in the proximal environment can significantly impact the development of social, such as young children’s peer interactions. Secondly, it is crucial to highlight positive role of teachers and continuously improving their professional competence. Teachers should provide emotional support to young children during educational activities, such as interactive games and classroom discussions to enhance their peer interaction skills. Thirdly, parents’ ability to educate and raise their children could be influenced by various factors, such as their educational level. Thus, teachers can leverage their professional strengths to offer appropriate guidance to parents and assist them in adopting positive parenting approaches while raising their children.

## 6 Limitations and further directions

First, regarding variable selection, it is worth noting that parental psychological control may affect many different aspects of young children’s social development. However, this study focused solely on young children’s peer interactions. Subsequent studies can further verify the correlation between parental psychological control and the social cognition, social–emotional, self-awareness, and self-management of young children. Apart from the emotional support provided by teachers that this study focused on, there may be other factors relating to teacher-child relationship and quality of teacher-child interactions, which could serve as moderating variables between parental psychological control and young children’s peer interactions. These factors need further verification and analysis in subsequent studies.

Second, concerning sample selection, this research selected children, parents, and classroom teachers from 5 kindergartens in a specific region of China using the convenience sampling principle, which has limitations on the sample size and distribution. In future research, modifications can be made to the sample selection to ensure broader applicability of the findings.

Third, in terms of research design, this study was cross-sectional and, therefore, it was unable to determine the causal relationship between variables. Future studies may attempt longitudinal follow-up studies to explore more definitive causal relationships between variables.

## Data availability statement

The original contributions presented in the study are included in the article/supplementary material, further inquiries can be directed to the corresponding author.

## Ethics statement

The studies involving humans were approved by the Ethical Committee of Northeast Normal University. The studies were conducted in accordance with the local legislation and institutional requirements. Written informed consent for participation in this study was provided by the participants’ legal guardians/next of kin.

## Author contributions

RC: Writing – original draft, Writing – review & editing. SL: Writing – original draft, Writing – review & editing. SH: Writing – original draft, Writing – review & editing. JY: Writing – original draft, Writing – review & editing.
